# Oligopeptide-modified poly(beta-amino ester)s-coated AdNuPARmE1A: Boosting the efficacy of intravenously administered therapeutic adenoviruses

**DOI:** 10.7150/thno.40902

**Published:** 2020-02-03

**Authors:** Pau Brugada-Vilà, Anna Cascante, Miguel Ángel Lázaro, Cristina Castells-Sala, Cristina Fornaguera, Maria Rovira-Rigau, Lorenzo Albertazzi, Salvador Borros, Cristina Fillat

**Affiliations:** 1 Institut d'Investigacions Biomèdiques August Pi i Sunyer (IDIBAPS), Centro de Investigación Biomédica en Red de Enfermedades Raras (CIBERER), Facultat de Medicina i Ciències de la Salut. Universitat de Barcelona, 08036 Barcelona, Spain; 2 Sagetis Biotech SL, 08017 Barcelona, Spain; 3 Grup d'Enginyeria de Materials (GEMAT) Institut Químic de Sarrià (IQS) Universitat Ramon Llull (URL), 08017 Barcelona, Spain; 4 Institute for Bioengineering of Catalonia (IBEC), The Barcelona Institute for Science and Technology (BIST), Barcelona, Spain; 5 Department of Biomedical Engineering and the Institute for Complex Molecular Systems, Eindhoven University of Technology, Eindhoven, the Netherlands

**Keywords:** oncolytic adenovirus, polymer-coated viral vectors, poly(β-amino ester)s, pancreatic cancer, systemic delivery

## Abstract

Oncolytic adenoviruses are used as agents for the treatment of cancer. However, their potential is limited due to the high seroprevalence of anti-adenovirus neutralizing antibodies (nAbs) within the population and the rapid liver sequestration when systemically administered. To overcome these challenges, we explored using nanoparticle formulation to boost the efficacy of systemic oncolytic adenovirus administration.

**Methods**: Adenovirus were conjugated with PEGylated oligopeptide-modified poly(*β*-amino ester)s (OM-pBAEs). The resulting coated viral formulation was characterized in terms of surface charge, size, aggregation state and morphology and tested for anti-adenovirus nAbs evasion and activity in cancer cells. *In vivo* pharmacokinetics, biodistribution, tumor targeting, and immunogenicity studies were performed. The antitumor efficacy of the oncolytic adenovirus AdNuPARmE1A coated with OM-pBAEs (SAG101) in the presence of nAbs was evaluated in pancreatic ductal adenocarcinoma (PDAC) mouse models. Toxicity of the coated formulation was analyzed *in vivo* in immunocompetent mice.

**Results**: OM-pBAEs conjugated to adenovirus and generated discrete nanoparticles with a neutral charge and an optimal size. The polymeric coating with the reporter AdGFPLuc (CPEG) showed enhanced transduction and evasion of antibody neutralization *in vitro*. Moreover, systemic intravenous administration of the formulation showed improved blood circulation and reduced liver sequestration, substantially avoiding activation of nAb production. OM-pBAEs coating of the oncolytic adenovirus AdNuPARmE1A (SAG101) improved its oncolytic activity *in vitro* and enhanced antitumor efficacy in PDAC mouse models. The coated formulation protected virions from neutralization by nAbs, as antitumor efficacy was preserved in their presence but was completely lost in mice that received the non-formulated AdNuPARmE1A. Finally, coated-AdNuPARmE1A showed reduced toxicity when high doses of the formulation were administered.

**Conclusions**: The developed technology represents a promising improvement for future clinical cancer therapy using oncolytic adenoviruses.

## Introduction

Oncolytic adenoviruses control tumor progression when locally administered through their oncolytic life cycle and activation of anti-cancer systemic immune responses, and several candidates have been demonstrated to be clinically relevant 1. However, their therapeutic efficacy is drastically limited when intravenously administered due to the rapid sequestration of circulating viral particles by the liver 2,3, widespread pre-existing immunity 4-7, and the induction of a strong innate immune response 8-11. To fully unlock the therapeutic potential of oncolytic adenoviruses, there is a need to develop strategies that bypass these hurdles and to boost their intravenous administration.

Strategies to achieve systemically injectable adenovirus (Ad) include replacing the Ad5 capsid with non-prevalent human serotypes 12, the use of the chimeric Ad5/Ad3 13 or Ad5/Ad48 14, genetic modifications in the capsid 15 and/or the physicochemical modification of Ad particles 16,17. Alternative strategies have focused on the use of biologically-derived microparticles 18 or extracellular vesicles 19,20.

Generation of hybrid vectors based on the surface modification of the Ad with a non-viral system, such as polymers or liposomes, has been widely explored. Conjugation of Ad with polyethylene glycol (PEG) via amine-mediated covalent bonding was reported to shield antibody binding sites, thereby preventing anti-Ad neutralization 21. However, PEGylation reduces CAR-mediated endocytosis entry, leading to low transduction efficiency 22. Other synthetic polymers, such as the poly-N-(2-hydroxypropyl)methacrylamide (polyHPMA), have also been used in a similar approach but again, the coating prevents cellular uptake 23. In both examples, transduction was restored by covalently attaching retargeting agents, such as FGF2 or EGF 24-27.

Engineering of Ad surfaces using ionically interacting polymers has also been demonstrated to improve the systemic delivery of virotherapeutic agents. Cationic polymers, such as poly(L-lysine), have been used in combination with PEG to ionically coat Ad vectors. Further, these have low toxicity and biodegradable properties, and evade nAbs neutralization while maintaining viral infectivity 28,29. HPMA copolymer has also been modified with oligolysine to produce an ionically interacting coating agent able to mediate CAR-cell transduction and to protect against antibody neutralization 30. However, cationic moieties have been associated with downstream toxicity 31.

Poly(β-amino esters) (pBAEs) are a class of biocompatible and biodegradable polymers that have recently emerged as promising gene delivery agents. They are composed of ester bonds and are easily synthesized by Michael addition of primary amines to diacrylates. Different types of pBAEs have been successfully used in several therapeutic applications, including vaccination 32, gene therapy for cancer and ophthalmology 33-35, gene silencing 36,37, and stem cell modification 38,39. Recently, our group has demonstrated that oligopeptide end-modified pBAEs (OM-pBAEs) have great potential as gene delivery vectors in terms of transfection efficiency, biocompatibility, and cell specificity *in vitro* 40,41*.* Moreover, we have expanded the use of this oligopeptide end-modification through the use of mixtures of different OM-pBAE polymers as delivery systems for siRNA and mRNA, leading to a simple method to tailor the surface charge of the resulting nanoparticles, while maintaining their ability to mediate efficient gene silencing 41,42. Further modifying the backbone polymeric structure to add an aliphatic amine chain (such as hexylamine) results in an optimized hydrophilic/hydrophobic ratio and increases the polyplex-cell affinity to biological lipid membranes, thereby improving their stability in physiological conditions 43. Overall, this modification allows for a safe and efficient *in vivo* administration 42.

Here we investigated whether a polymeric coating based on a formulation of modified OM-pBAEs overcomes the limitations associated with systemic delivery of Ads, and if such approach improves the efficacy and safety of oncolytic adenoviral therapy. We found that OM-pBAEs-modified Ad had improved circulation lifetime and decreased interactions with antibodies, with less liver tropism and a lower innate immune response. Notably, coating the oncolytic AdNuPARmE1A revealed enhanced anti-cancer efficacy in pancreatic tumors and the capacity to avoid nAbs *in vivo.*

## Results and Discussion

### Generation and biophysical characterization of OM-pBAE-Ad nanocomplex

**Figure 1:**
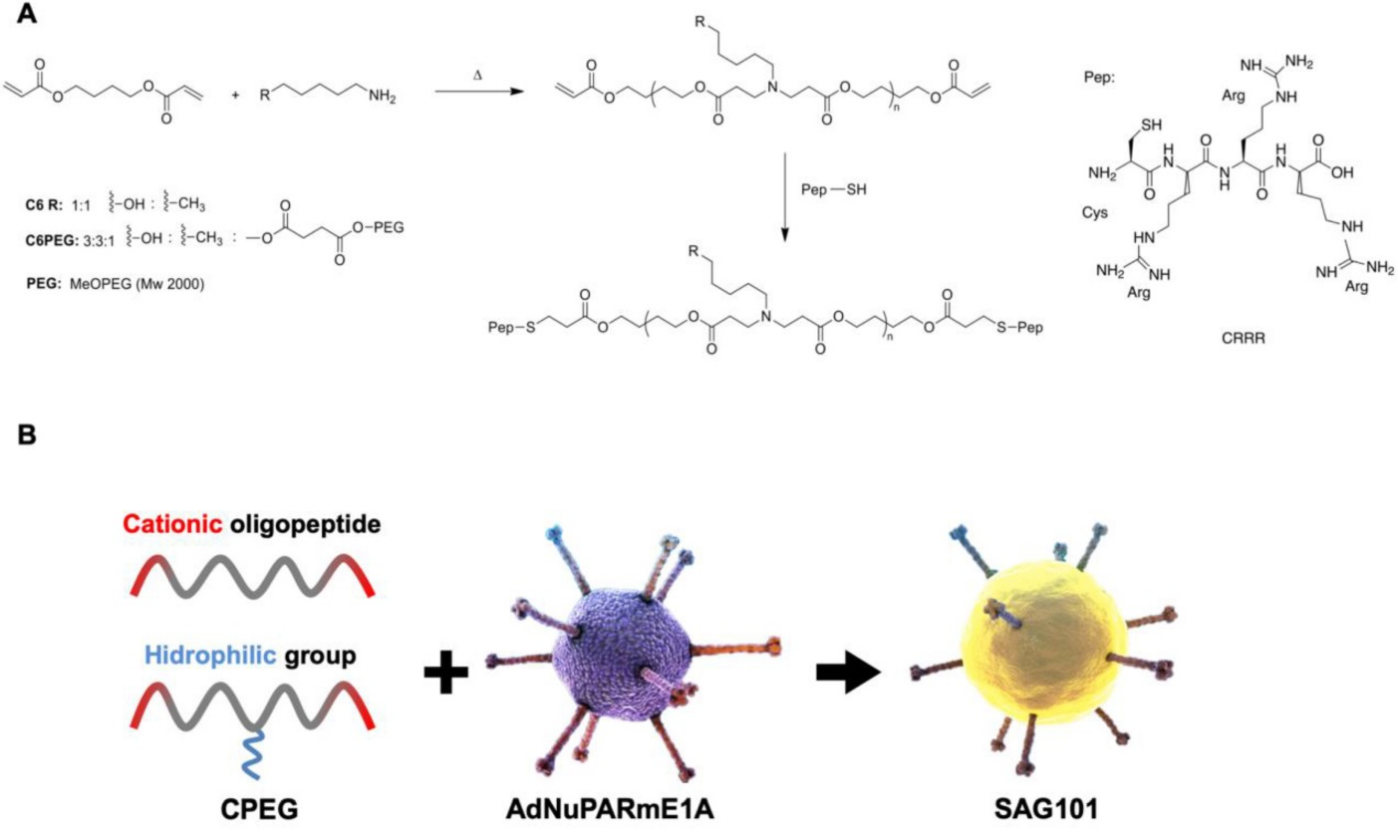
** Characteristics of the polymers and oncolytic adenovirus coating. A** Synthesis steps and chemical structure of C6CR3 and C6PEGCR3.** B** Schematic representation of the OM-pBAEs-based coating technology for therapeutic oncolytic adenoviruses.

To obtain efficient coating of viral particles, we synthesized two different polymers following a Michael addition of primary amines to diacrylates, as described in Figure 1A. The C6CR3 polymer contained three types of monomers (1,4-butanediol diacrylate, 5-amino-1-pentanol and 1-hexylamine) and an oligopeptide composed of one cysteine residue and three arginine residues (CR3) at both ends. The C6-PEG-CR3 polymer was synthesized by combining the three monomers with a fourth one containing a PEG moiety; the same terminal oligopeptide (CR3) was added to both termini of this second backbone. Both polymers were combined with adenoviral particles to coat them by electrostatic interactions between the negatively-charged virus surface and the positively-charged polymer chains (Figure 1B).

**Figure 2:**
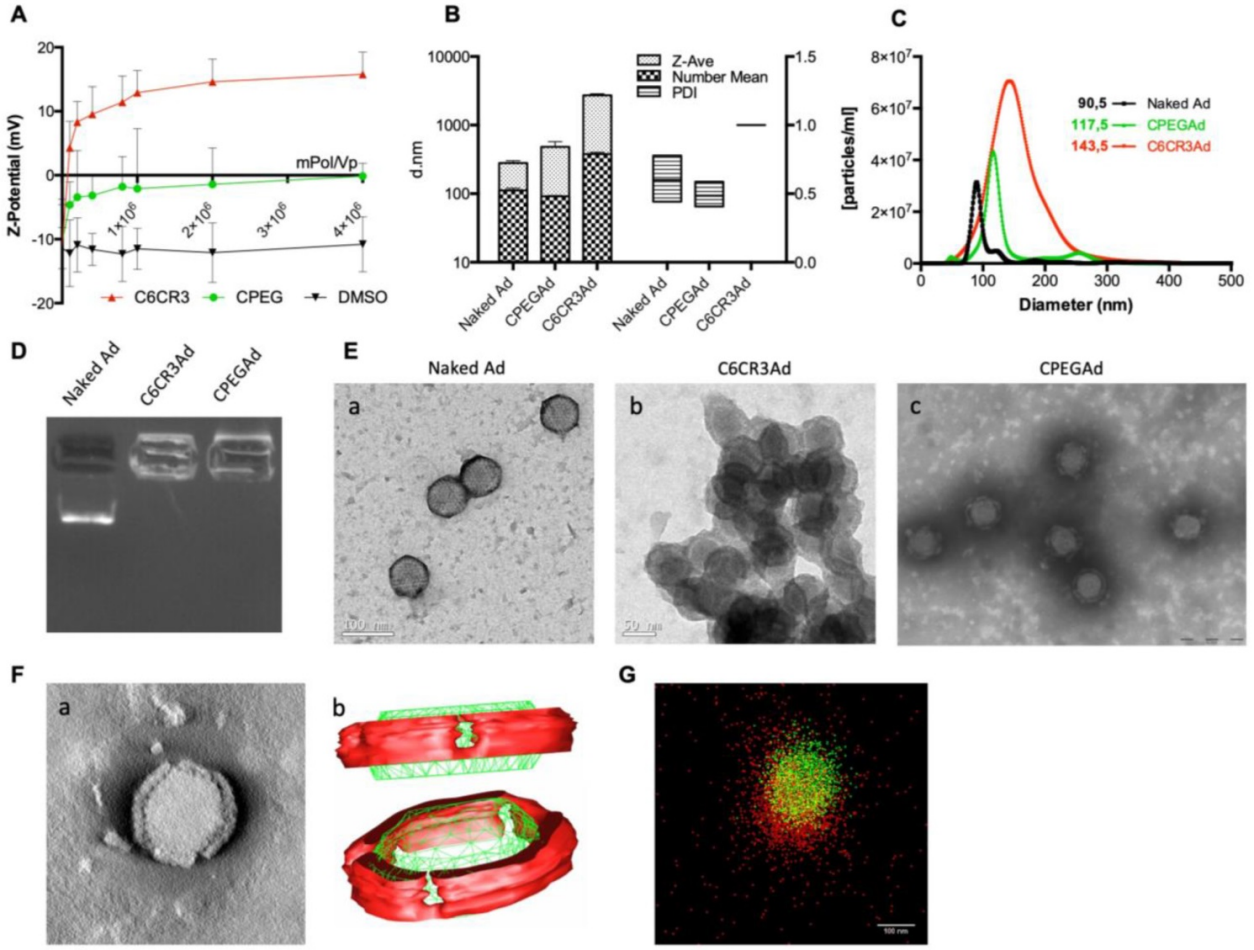
** Biophysical characterization of OM-pBAE-adenovirus complexes. A** Z-potential determination of viral particles complexed with increasing ratios of molecules of polymer/vp for C6CR3 and CPEG polymers, or (as a control) with the DMSO vehicle without polymer. **B** Size determination by DLS. Z-Average, mean number, and polydispersity index (PDI) are presented. **C** Size distribution determined by NTA of naked Ad, CPEGAd, or C6CR3Ad formulations. **D** Encapsulation capability of OM-pBAEs assessed by gel-retardation assay. **E** Transmission electron microscopy (TEM) images of negatively stained formulations. TEM image of naked Ad (a), C6CR3-coated Ad (b), and CPEG-coated Ad (c). Scale bar: 100 nm for a; 50 nm for b and 200 nm for c. **F** Single CPEGAd particle analysis by TEM tomography. TEM tomography frame (a), three-dimensional representation of the resulting tomography constructed using 3DMOD software. The red solid object represents the coating and the green structure represents the contour of the viral particle (b). **G** dSTORM characterization of fluorescently labeled CPEGAd complexes. Green signal from DyLight550 labeled viral particles and red light from Cy5-labeled pBAE molecules.

The physicochemical properties of the adenovirus (AdGFPLuc) coated with the C6CR3 polymer (termed C6CR3Ad) or with a mixture of C6CR3:C6PEGCR3 2K (65:35) polymers (CPEG) (termed CPEGAd) were characterized as compared to the naked adenovirus. Ad particles (1 × 10^10^ vp) were mixed with increasing concentrations of the polymers, and the surface charges were analyzed by DLS measuring the Z-potential. Changes in the surface charge of coated particles reached a plateau at 1 × 10^6^ molecules of polymer/vp. The C6CR3 nanoparticles had a positive charged, whereas the CPEGAd nanoparticles had a neutral charge, demonstrating the coating of the negatively charged adenoviral particles through electrostatic interactions (Figure 2A). The ratio of 4 × 10^6^ molecules of polymer/vp was chosen to ensure that an excess of polymer interacted with viral capsids. The mean size of the CPEG-coated Ad (99.5 nm) was very close to that of the naked Ad (108.5 nm) but was about 7× higher for C6CR3Ad (700.9 nm). Likewise, CPEGAd had Z-average values and polydispersity index (PDI) (481 ± 158 nm, PDI = 0.485 ± 0.092) that were similar to those of naked virus particles (279.5 ± 38 nm, PDI= 0.599 ± 0.16), whereas C6CR3Ad showed a significant increase in the Z-average size (C6CR3Ad ˃ 1000 nm) and had a PDI=1, clearly indicating the presence of aggregates. To accurately measure size increase without being influenced by the presence of aggregates (in the case of C6CR3Ad), NTA measurements were performed (Figure 2B). NTA analysis revealed a unique peak for each formulation of mean size of 90.5 nm for naked Ad, 117.5 nm for CPEGAd, and 143.5 nm for C6CR3Ad; notably, the sharpness of the peaks also varied between formulations, with a much wider peak for C6CR3Ad (Figure 2C). These results suggest that the presence of PEG in the formulation helped to form more homogeneous colloidal suspensions, with aggregates forming in its absence. To further demonstrate the change in surface charge and size of polymer-coated viral particles, we performed gel retardation assays, which revealed the abrogation of migration of coated virus particles towards the positive pole (Figure 2D).

In agreement with the results obtained by DLS and NTA, transmission electron microscopy (TEM) imaging confirmed the capacity of both polymers to interact with viral capsid surfaces, and to form aggregates in the C6CR3 complexes but not in the CPEG complexes, indicating that the presence of PEG in the formulation resulted in disperse coated particles (Figure 2E). Moreover, TEM tomography showed a well-defined coating around the viral particles, with a mean thickness of 15.1 ± 2.2 nm (Figure 2F a). Non-coated regions were observed in the penton base from which the fiber protein extends and were confirmed by three-dimensional representation (Figure 2F b). Finally, further evidence of the polymer-viral particle interaction in solution was demonstrated by direct stochastic optical reconstruction microscopy (dSTORM) analysis. Polymer and adenovirus particles fluorescently labeled with Cy5 and DyLight 550, respectively, were mixed and imaged by dSTORM. Cy5 and DyLight 550 signals were pseudo-colored red and green, respectively. Appreciable amounts of colocalized signal (yellow) was observed in CPEG formulations (Figure 2G). The stability of polymer-virus complexes under physiological conditions was analyzed by comparing the transduction efficiencies of naked AdGFPLuc and CPEGAd over time 44. GFP fluorescence of PANC-1 cells transduced with the naked virus rapidly declined, with only 20% of GFP-positive cells detected after 48 h although 70% of cells expressed GFP in CPEGAd cultures (Figure S1). These results suggest that CPEG formulation protects the Ad particles. Overall, these results demonstrate that CPEG polymer successfully complexed with viral particles, and that the formulation resulted in disperse nanoparticles with reasonably increased size, a neutral charge, and relatively good stability.

### CPEG coating protects adenovirus from neutralizing antibodies and improves cancer cells transduction *in vitro*

**Figure 3:**
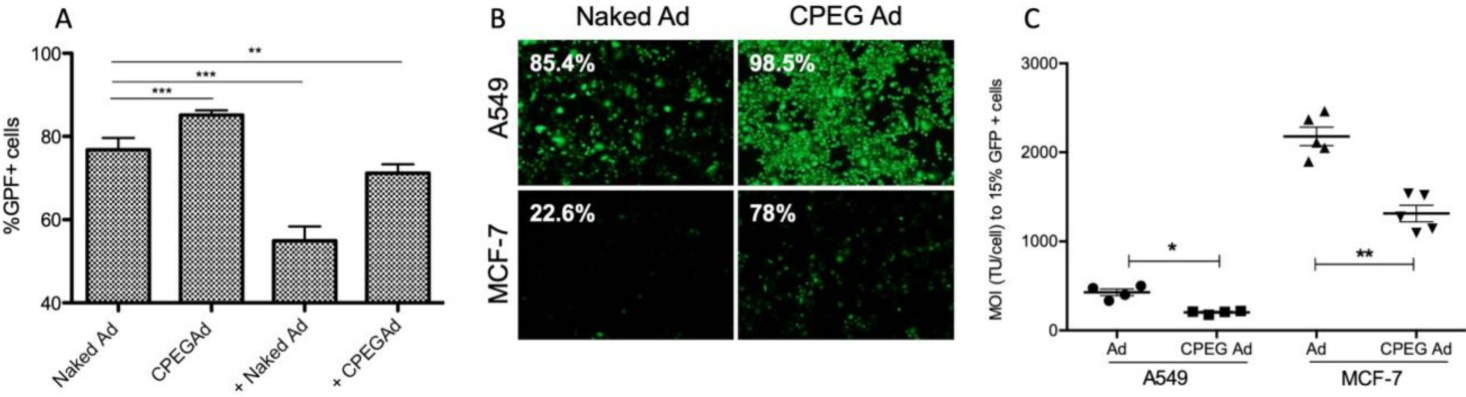
** Transduction efficiency of CPEGAd in the presence of NAbs and CAR-independent cellular uptake. A** Flow cytometry quantification of PANC-1 GFP-positive cells after infection with naked or CPEG-coated GFP-expressing reporter AdGFPLuc at MOI 50 in the presence or absence of nAbs. The positive symbol (+) represents pre-incubation of virus samples with 1 × 10^4^ dilution of commercial anti-Ad5 antibody (ab6982, Abcam). **B** Representative fluorescent images of A549 and MCF-7 cells transduced at MOI 100 of naked Ad or CPEGAd. The percentage of GFP-positive cells resulting from flow cytometry assay is indicated. **C** Infectivity of CPEG-coated AdGFPLuc in CAR+ (A549) and CAR- (MCF-7) cells. Five thousand cells/well were infected with naked Ad and CPEGAd at increasing MOIs ranging from 0 to 4500, and the percentage of GFP-positive cells was analyzed by flow cytometry. The MOI needed to achieve 15% of GFP-positive cells was determined and is shown for each condition. *P < 0.05, ** P < 0.01, *** P < 0.001

Efficient shielding of the Ad particles is essential to prevent elimination of the virus by the immune system. In this respect, we studied whether the CPEG complexes could protect the virus from neutralizing antibodies (nAbs) *in vitro*. Pancreatic cancer PANC-1 cells were infected with 50 MOI of naked AdGFPLuc (naked Ad) or coated CPEGAd in the absence or presence of a 10,000-fold dilution of a commercial neutralizing antibody (Ab6982) against Ad5. The presence of nAbs in the cultures infected with the naked Ad reduced the transduction efficiency from 78% to 55%. The CPEGAd coated formulation remarkably enhanced viral transduction up to 71% in the presence of nAbs, indicating that the coating protected the virus. Interestingly, in the absence of nAbs, the coated CPEGAd virus increased cellular transduction up to 85% (Figure 3A).

As cell-penetrating peptides containing arginine residues have been shown to transfer nucleic acids efficiently to the cell 45 and Ad modified with arginine polymers have enhanced transduction in a CAR-independent pathway 46, we investigated whether the CPEGAd formulation could facilitate transduction through CAR-independent cellular uptake. To this end, CAR-positive (CAR+) A549 cells and CAR-negative (CAR-) MCF7 cells were infected with the naked reporter adenovirus AdGFPLuc or the CPEGAd formulation at MOIs ranging from 0 to 4500 TU/cell, and GFP-positive cells were visualized and quantified by flow cytometry after 48 h. The transduction efficiency of CPEGAd was markedly increased compared to the naked Ad in both A549 and MCF-7 cells (Figure 3B). The increased infectivity of the CPEGAd formulation was also observed both in A549 and MCF-7 cells, by the statistically significant lower MOI needed to reach 15% of GFP-positive cells (Figure 3C). Of note, A549 CAR+ cells showed much higher CPEGAd transduction than MCF-7 cells, suggesting that CAR-mediated uptake was also active for the CPEGAd (Figure 3B). However, a significant increase in GFP-positive cells was also observed in MCF-7 cells when they were transduced with CPEGAd, suggesting that the CPEG formulation facilitates Ad transduction and that this improvement is at least in part independent of CAR expression. Similar to what has been proposed for other coating strategies, it is very likely that the CPEGAd complex could enter the cells via a CAR-independent entrance through caveolae- or micropinocytosis-mediated endocytosis. Further, via a CAR receptor-mediated endocytosis, the neutral charge of the formulation could facilitate physical contact between viral particles and cell membrane enhancing CAR-mediated uptake 47.

### Systemic administration of CPEGAd improves blood persistence and reduces liver sequestration

**Figure 4:**
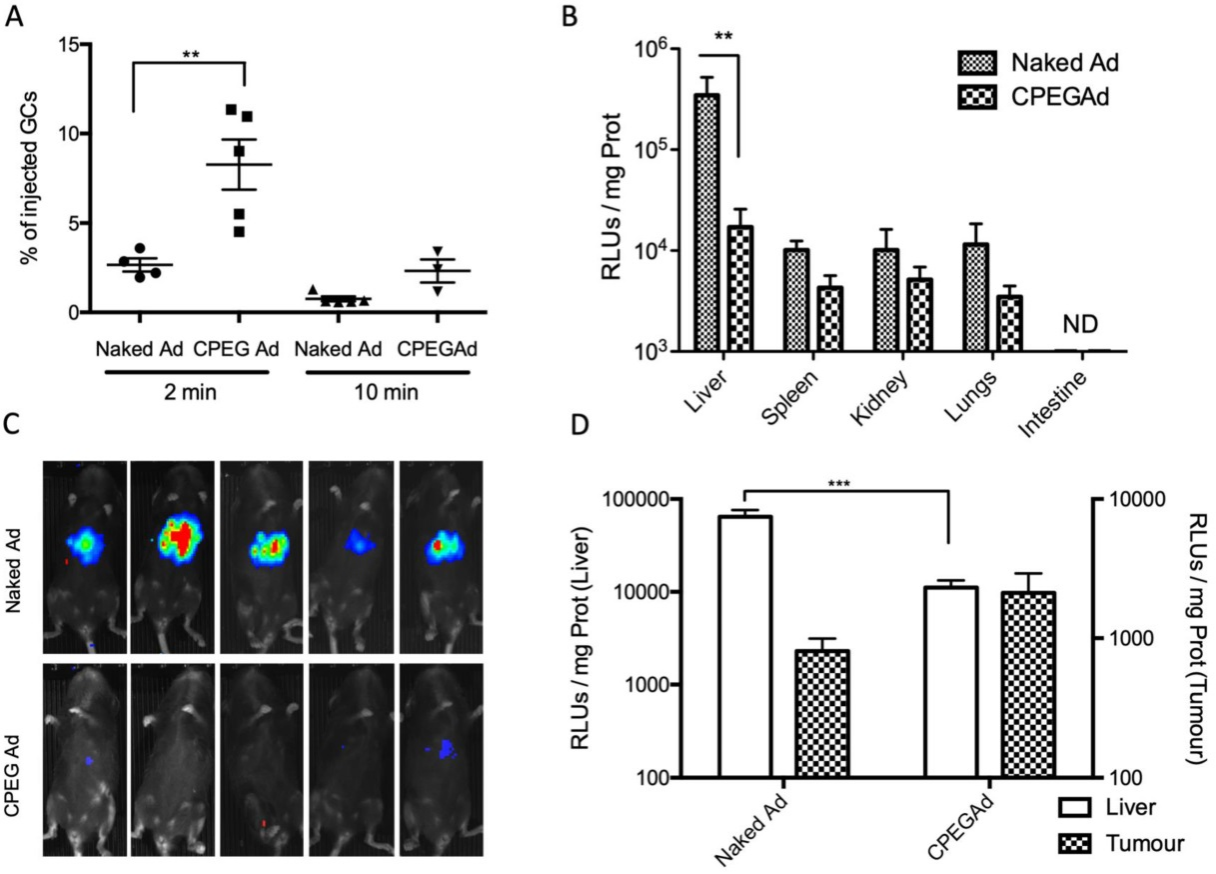
** Circulation kinetics and biodistribution of CPEGAd *in vivo*. A** qPCR quantification of virus genome copies (GCs) in blood at 2 or 10 min after intravenous administration of 1 × 10^10^ AdGFPLuc vp/animal in C57BL/6J mice of naked and CPEG-coated formulation (n = 5). **B** Luciferase activity quantification of protein extracts from liver, spleen, kidneys, lungs, and intestine of treated mice at five days after injection. **C** Bioluminescence *in vivo* imaging of mice at 5 days after injection. **D** Tumor-to-liver *in vivo* transduction study of CPEGAd complexes in PANC-1 tumor-bearing BALB/c Nu/Nu mice (n = 6) in each flank. Luciferase activity of protein extracts was quantified five days after the iv administration of 1 × 10^10^ vp/animal of naked AdGFPLuc and CPEGAd. *P < 0.05, **P < 0.01, ***P < 0.001

The half-life of Ad in blood has been estimated to be less than 2 min, due to the rapid liver sequestration of Ad by Kupffer cells (KC) 48. PEGylation has been shown to mitigate clearance by KC, although the PEG size seems to be crucial 22. Moreover, some polymer-coated viruses have shown increased blood circulation half-live 22,49. To study the blood persistence of the CPEGAd formulation and to compare it to that of naked Ad, we intravenously injected 1 × 10^10^ vp of naked Ad or the CPEGAd formulation into C57BL6/J mice, and the presence of virus genomes were analyzed at 2 min and 10 min after injection by qPCR. Interestingly, a statistically significant 2.3-fold increase in the number of genome copies was detected for CPEGAd complexes 2 min post-administration. Moreover, at 10 min post-administration, only 0.75% of the injected naked virus but 2.71% of the CPEGAd was detected, indicating that the coated virus has a prolonged circulation time (Figure 4A).

We next studied the tissue biodistribution of the systemically injected naked and CPEG formulated adenovirus. Five days after viral particles administration, animals were imaged *in vivo*, and organs were extracted from euthanized animals to analyze luciferase activity in tissue extracts. A robust liver detargeting effect was observed both by whole animal bioluminescence imaging and by quantification of luciferase activity in individual organs (Figure 4B-C). Spleen, kidney, and lungs also showed a reduced transduction with the formulated virus. These results suggest that physical masking of the HEXON protein in the Ad surface with OM-pBAEs could minimize interaction with Factor X, thus minimizing liver transduction.

Higher levels in blood circulation and the significant liver detargeting indicated higher bioavailability of systemically administered Ad, which could improve its accumulation in solid tumors. In these studies, any potential interference of Ad binding to erythrocytes that could compromise Ad bioavailability was not considered, as the experiments were conducted in mice, in which Ad have been shown to have negligible interactions with erythrocytes 50. The scenario could be different in humans, as Ad5 binds to human erythrocytes through the CAR receptor 51. However, recent reports point that this interaction does not internalize the virus, due to the lack of integrins, and results in a transient sequestration of Ad by erythrocytes, suggesting that human erythrocytes do not represent a major obstacle for systemic delivery 52.

To determine if there could be the potential benefits of CPEGAd tumor targeting *in vivo*, nude mice with subcutaneous PANC-1 tumors were intravenously injected with 1 × 10^10^ vp of naked Ad or the CPEGAd formulation, and luciferase activity in the tumors and the livers was quantified after 5 days. Although not statistically significant, increased tumor transduction was observed with the CPEG virus, with a mean value of 2116 RLUs/mg as compared to 808 RLUs/mg with the naked virus. In this *in vivo* model, we could also confirm a liver detargeting effect of the coated formulation (Figure 4D).

### Oncolytic CPEG-coated adenovirus displays enhanced cytotoxicity and maintains its anti-tumor activity in pre-immune mice after systemic administration, with a good safety profile

To analyze the potential therapeutic value of the coating formulation, we complexed the CPEG polymers with the oncolytic adenovirus AdNuPARmE1A, previously shown by our group to efficiently suppress pancreatic tumor growth 53. Tumor selectivity of this Ad is achieved by replacing the E1A wild-type promoter with a chimeric promoter containing *uPAR* gene regulatory sequences and a stretch of binding sites that confer Notch-dependent activation to the promoter. First, we analyzed whether the formulation of AdNuPARmE1A with CPEG (termed SAG101) resulted in improved viral infectivity as compared to the naked AdNuPARmE1A (termed AdNu). To this end, cells were infected at serial virus dilutions, and the number of infective viral particles was analyzed by HEXON immunostaining. SAG101 resulted in a 3.3-fold increase in the number of PFU/ml indicating enhanced infectivity, similar to what was observed with the reporter adenovirus CPEGAd when compared to naked Ad (Figure S2).

**Figure 5:**
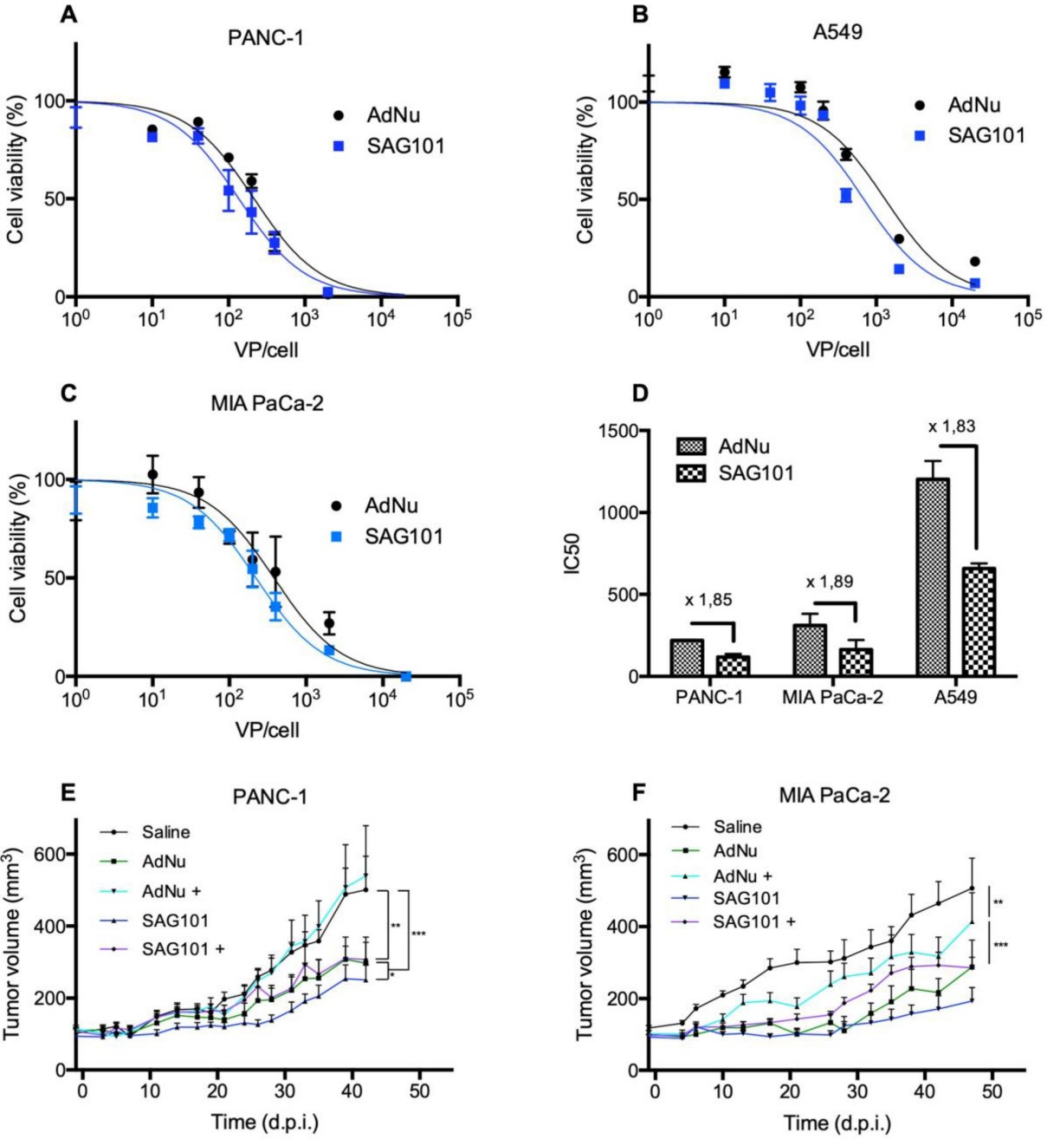
** Antitumoral efficacy of SAG101 (CPEG-coated AdNuPARmE1A) *in vitro* and *in vivo*. A-C** Dose-response curves of PANC-1, MIA PaCa-2, or A549 tumoral cell lines treated with SAG101 or AdNu, with doses ranging from 1 to 20,000 vp/cell. Cell viability was quantified by MTT assay three days after infection. **D** IC_50_ summary graph including the fold-change between coated and naked formulations. Results are shown as mean ± SEM of three independent experiments performed in triplicate for each condition. **E, F** Efficacy studies of SAG101 and AdNuPARmE1A in passively immunized PANC-1 and MIA PaCa-2 tumor-bearing mice (n = 8). Animals of the pre-immune groups (marked as + conditions) were passively-immunized by I.P. injection of neutralizing serum the day before the treatment. Eight animals per group were treated with 4 × 10^10^ vp/animal. Tumor progression data were compared between conditions using linear mixed effect in R v2.14.1 with the lme4 package. *P < 0.05, ** P < 0.01, *** P < 0.001

Next, we analyzed the cell-killing effect of SAG101 on PANC-1, MIA PaCa-2, or A549 cells. Cells were infected with increasing virus doses, and cell viability was analyzed three days later. Dose-response curves were analyzed (Figures 5A-C), and IC_50_ values were calculated. A 1.8-fold decrease in the IC_50_ with SAG101 compared to AdNu was observed in all tested cell lines (Figure 5D).

The antitumor efficacy of SAG101 *in vivo* was studied in PANC-1 and MIA PaCa-2 pancreatic xenograft mouse models after systemic delivery in naïve or anti-Ad5 pre-immunized mice. To trigger a pre-immune condition in nude mice, we passively immunized them by an intraperitoneal injection of neutralizing sera from C57BL6/J mice previously treated with naked wild-type Ad5. The neutralization capacity of the sera was tested *in vitro* in a dose-response neutralization assay. Sera showed equivalent neutralization capacity in the efficacy studies of the two tumor models (Figure S3). Naïve or passively-immunized mice bearing PANC-1 and MIA PaCa-2 tumors received 5 × 10^10^ vp of AdNu or SAG101 intravenously and tumor growth was monitored over time. In naïve PANC-1 tumor-bearing mice, a statistically significant improvement of the SAG101 efficacy with respect to AdNu treatment was observed with a final tumor growth inhibition (TGI %) of 61.5% for SAG101 and 52.1% for AdNu. This effect could be attributed to the enhanced infectivity, improved pharmacokinetics, and/or the liver detargeting of SAG101. In contrast, AdNu was completely inefficacious in pre-immunized mice (-9.9 % TGI), in contrast to SAG101, which showed an antitumor activity equivalent to naïve AdNu treatment (49.2 % TGI). MIA PaCa-2 tumor-bearing mice showed similar results, although the neutralization of AdNu in pre-immunized mice was not as efficient as observed for the PANC-1 tumor-bearing experiment (Figure 5C). These results confirm that nAbs efficiently block oncolytic adenovirus activity and demonstrate that CPEG polymer coating effectively mediates protection of Ad against nAbs.

**Figure 6:**
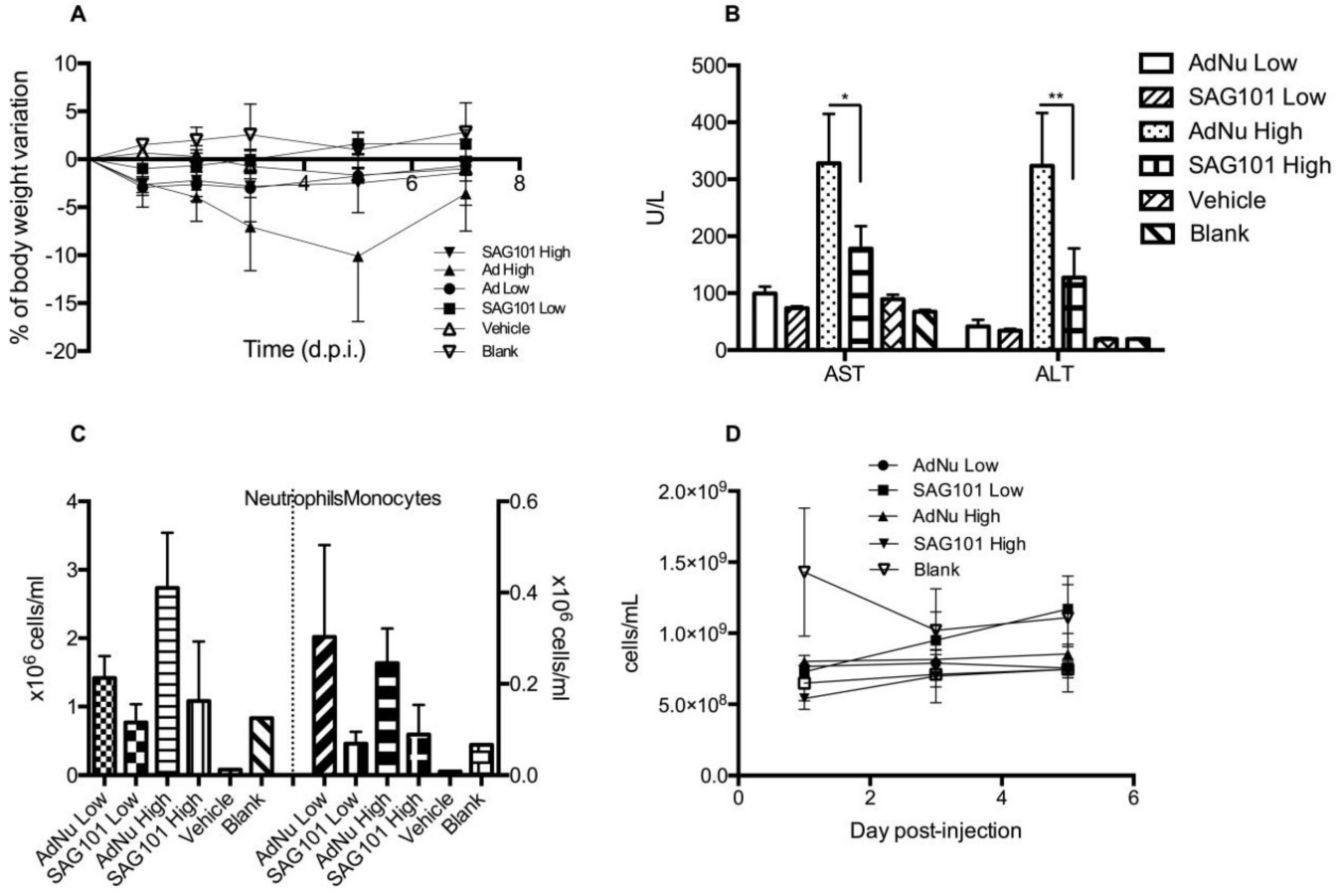
** General toxicity profile of SAG101 after systemic administration in immunocompetent mice. A** Percentage of body weight variation of BALB/c mice (n = 8, n = 3 for controls) after intravenous administration of two different doses of AdNu and SAG101 (low, 5 × 10^10^ vp; or high, 7.5 × 10^10^ vp); the same amount of vehicle was injected at a high virus dose. **B** Assessment of hepatotoxicity based on AST and ALT in the serum of treated mice at 7 days post-administration. Results are expressed as the mean ± SEM of n = 8 animals/group, or n = 3 for the control groups. *P < 0.05, ** P < 0.01. **C** Peripheral blood cell counting of neutrophils and monocytes in response to AdNu or SAG101 intravenous injection. **D** Platelet cells counts of BALB/c peripheral blood at the indicated time-points after treatment. Data show mean values ± SEM.

To further investigate the potential of SAG101 therapy, we assessed its safety profile *in vivo* in immunocompetent mice. BALB/c animals were intravenously injected with vehicle, AdNu, or SAG101 at a low or high virus dose (Figure 6). Body weight, liver enzymes levels (AST and ALT), and hematological parameters were analyzed. At day 5 post-administration, animals treated with AdNu at the highest dose reached a maximum body weight loss of 10.1 ± 6.8%. All the other groups showed a similar weight loss of around 2% (Figure 6A). Furthermore, the highest increase in AST and ALT levels was also observed in animals treated with the highest dose of AdNu, even though the levels were moderate (Figure 6B). Increased monocytes and neutrophils were observed with the non-coated virus when compared to vehicle or polymer alone. Interestingly, there was a reduced induction of monocytes and neutrophils with SAG101 (at either low or high dose), suggesting it protects against the activation of innate immune responses (Figure 6C-D). A mild thrombocytopenia was detected in all virus-injected groups at one day after the administration, although animals injected with SAG101 at the low dose showed a faster recovery rate, reaching normal platelet counts at three days after injection (Figure 6E). In sum, in all the parameters tested, SAG101 (coated AdNuPARmE1A) improved the toxicity profile as compared to the naked oncolytic virus.

### CPEG polymers reduce *de novo* production of neutralizing antibodies against adenovirus and mitigate innate immune responses

**Figure 7:**
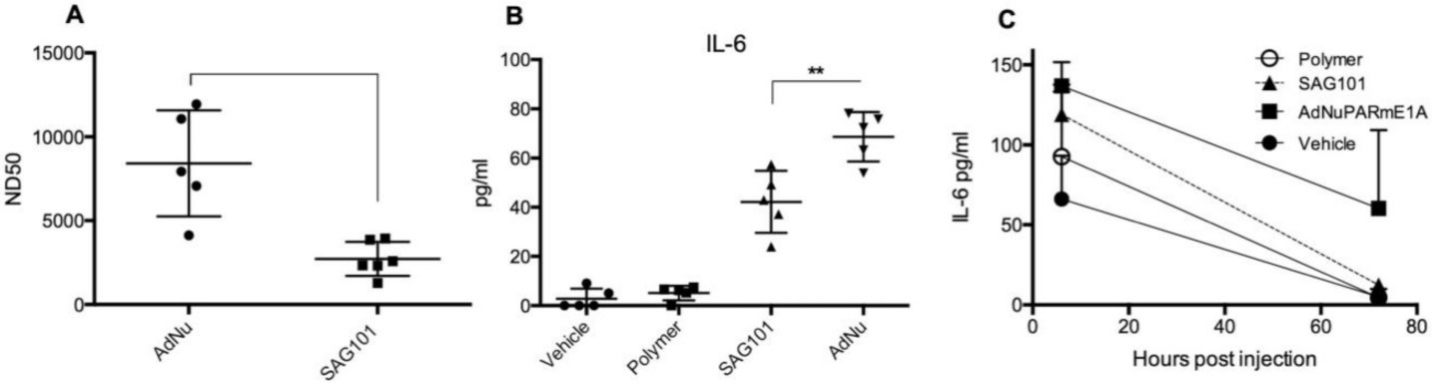
** Immune response against OM-pBAE coated adenovirus *in vitro* and *in vivo*. A** Neutralizing capacity of serums collected from naïve C57BL/6J mice after intravenous administration of two doses of naked Ad and CPEGAd (1 × 10^10^ vp/animal) were administered at days 1 and 14; and sera were collected at day 21 post-injection. Resulting sera were analyzed in order to determine the ND50 (dilution needed to neutralize 50% the infectivity in an *in vitro* neutralization assay). **B** IL-6 production by RAW264.7 macrophages in response to exposure to AdNu and SAG101. Cytokines were quantified from culture media collected 48 h post-infection of 1 × 10^6^ cells/well treated with 1000 vp/cell of naked AdNu, SAG101, the polymeric component without viral particles, or (as a control) the corresponding volume of DMSO as in the SAG101 sample. **C** *In vivo* cytokine release study in immunocompetent BALB/c mice in response to AdNuPARE1A and SAG101 intravenous injection. Serum samples were collected at 6 h and 72 h after intravenous administration of 5 × 10^10^ viral particles of each formulation and the respective amount of polymeric component without viral particles as a control. IL-6 levels in serum was quantified using a microbead-based ELISA kit (mouse cytokine 10-Plex Panel; Invitrogen) on the Luminex^TM^ 200^TM^. Data show mean values ± SEM. *P < 0.05, **P < 0.01, ***P < 0.001

Our results indicate that the CPEG formulation helped adenovirus to escape from neutralization induced by anti-Ad5 specific antibodies, both *in vitro* and in *vivo* (Figures 3A, 5E, and 5F). We next investigated whether CPEG coating could also protect from the production of anti-adenovirus nAbs *in vivo*. To do so, C57BL6/J mice received an intravenous injection of 1 × 10^10^ vp from naked Ad or CPEGAd at day 1 and 14; sera obtained at day 21 were then tested an *in vitro* neutralization assay. Notably, CPEGAd formulation sera displayed a statistically significant lower neutralization capacity when compared to sera from mice treated with naked Ad (Figure 7A). These results indicate that the activation of adaptive immune response *in vivo* is diminished with the coating formulation and suggest that re-administration procedures of coated therapeutic adenovirus could be efficacious.

Administration of Ad particles systemically results in the initiation of strong innate immune responses through the rapid uptake of adenovirus by resident macrophages in the liver, that clear them from circulation while secreting several cytokines. To evaluate whether the polymeric coating had an impact on the induction of this innate immune response, we first studied the effects of the CPEG-complexed oncolytic adenovirus AdNuPARmE1A on IL-6 secretion in the murine macrophage cells RAW264.7. We observed that SAG101 infection induced a significant reduction in the release of IL-6 as compared to that of the naked virus. Interestingly, the CPEG polymer alone did not trigger any IL-6 release (Figure 7B). Next, we measured the cytokine release in mice that had been i.v. injected with 5 × 10^10^ vp of AdNuPARmE1A or SAG101. Serum levels of cytokine IL-6 analyzed at 6 h and 72 h showed a trend towards reduced induction of IL-6 with the coated virus, although the effects were not statistically significant. Notably, the IL-6 induction completely resolved at 72 h, in contrast to that observed with the naked virus (Figure 7C). Only a slight increase in IL-6 at 6 h was observed after *in vivo* administration of the polymer. These results demonstrate that masking the adenoviral particles with the CPEG formulation reduces the induction of the innate immune response.

Our results indicated that shielding the adenovirus capsid with a PEGylated OM-pBAEs polymer formulation can safely minimize the major barriers of systemic delivery, which indicates an antitumor efficacy that should encourage the use of oncolytic adenovirus systemic delivery in clinical applications.

Until now, oncolytic adenoviruses have been shown to have clinical efficacy when delivered intratumorally, by triggering cell lysis and eliciting an antitumor immune response (as virus treatment facilitates the infiltration of CD8+ T-lymphocytes that recognize specific tumor-antigens) 19,54,55. However, oncolytic adenovirus will need to be delivered systemically to be considered for a pan-cancer treatment, as this would permit the viruses to reach metastatic foci, where they could replicate and enhance the antitumor immune response. Due to the heterogeneous nature of tumors, the neoantigens in the primary tumors and the metastasis are not likely to coincide, thus the antitumor immune response generated in the primary tumor might not be efficacious in the metastatic foci. Our results reinforce the potential of using systemic oncolytic adenoviral delivery against both primary tumors and disseminated lesions.

## Conclusions

We have successfully developed a PEGylated OM-pBAE polymer formulation, named CPEG, that is able to complex with adenovirus. This coating technology produced neutral disperse nanoparticles. Coated adenoviral particles were protected from neutralization by pre-existing neutralizing antibodies. Systemic administration of the formulated adenovirus prolonged the circulation time, reduced the liver targeting, minimized the production of nAbs, and improved tumor tropism. The oncolytic adenovirus AdNuPARmE1A coated with CPEG polymers (termed here SAG101) displayed antitumor efficacy in pre-immunized mice, whereas non-modified AdNuPARmE1A was completely neutralized. Systemic administration of SAG101 induced negligible hepatotoxicity and reduced innate immune response.

Our results show that CPEG formulation improves the safety and effectiveness of oncolytic adenovirus therapy. We therefore propose SAG101 as a therapeutic agent with great potential against pancreatic adenocarcinoma.

## Materials and Methods

### Cell culture and adenoviruses generation

Human pancreatic tumor cell lines (PANC-1, MIA PaCa-2), lung cancer A549 cells, breast cancer MCF-7 cells, HEK293 E1-transcomplementing cells, and RAW 264.7 macrophages were obtained from the American Type Culture Collection (ATCC, Rockville, MD, USA) and cultured following the ATCC recommendations.

The recombinant adenoviruses used in this study (AdGFPLuc and AdNuPARmE1A) were previously described 53. Briefly, AdGFPLuc is a non-replicating adenovirus vector carrying enhanced green fluorescence protein (GFP) and luciferase (Luc). AdNuPARmE1A is a conditionally replicating adenovirus with strong oncoselectivity for pancreatic cancer cells. AdGFPLuc was amplified in HEK 293 E1-transcomplementing cells and AdNuPARmE1A was amplified in A549. Viruses were purified by cesium chloride density gradient centrifugation according to standard techniques 56. Quantification of resulting viral particle (vp) concentrations was determined by optical density measurements at 260 nm (OD260).

### Preparation of fluorescently-labeled Ad viral particles

Fluorescently-labelled adenoviruses were prepared using the DyLight 550 Microscale Antibody labeling Kit (ThermoFischer). The labelling reaction was carried out by preparing 100 μl of virus solution in PBS with 10 μl of borate buffer (0.67 M) at 1.3 × 10^12^ vp/ml. The virus solution was then mixed with the labelling reagent (provided as a powder in an Eppendorf tube) and incubated for 1 h at RT. Labelled virus was then washed 8 times using Microcon Centrifugal Filters 10K (Merck) with PBS++ in order to remove the excess of free fluorophore.

### Synthesis and characterization of OM-pBAEs

OM-pBAEs were synthesized as described previously 42 Briefly, addition reaction of primary amines (5-amino-1-pentanol and hexylamine), together with a methoxy-PEG-NH2 monomer in the case of the C6PEG polymer, to an excess of diacrylates (1,4-butanediol diacrylate) was used to synthesize an acrylate-terminated polymer. These polymers were then end-capped with oligopeptides composed of 1 Cys + 3 Arg (CRRR or CR3) in DMSO. Synthesized structures were confirmed by 1H NMR, recorded in a 400 MHz Varian (NMR Instruments, Claredon Hills, IL), using methanol-d4 as a solvent. Molecular weight (MW) relative to polystyrene standard was determined by HPLC (HPLC Elite LaChrom system of VWR-Hitech equipped with a GPC Shodex KF-603 column and THF as mobile phase).

### Labelling of OM-pBAEs with Cy5 fluorophore

Cyanine-5 NHS ester (ab146454; Abcam) amine-reactive red emitting fluorescent dye was used to react with the free amino group on the cysteine amino acid present in terminal peptides used for end-capping pBAEs backbones. The labelling reaction was done by preparing 0.5 ml of cysteine-5 NHS ester solution at 10 mg/ml in DMSO and mixed with C6CR3 (0.035 ml, 100 mg/ml, 0.97 μmol) in DMSO to a final volume of 0.8 ml. Triethylamine (0.004 ml, 29 μmol) was added to the solution. The tube was stirred (protected from light) at room temperature for 20 h. A 7:3 diethyl ether/acetone (1.5 ml) solution was then added dropwise to the mixture, the suspension was centrifuged at 4000 rpm for 10 min, and the supernatant was discarded. The solid was washed with 7:3 diethyl ether/acetone (0.6 ml) twice. The product was dried under vacuum, and the final solution was prepared by resuspending the solid in DMSO at 100 mg/ml.

### Preparation and characterization of OM-pBAE-coated adenoviruses

Coated virus samples were prepared by diluting the virus stock (VS) solution 1:50 in PBS. Taking into account the concentration of viral particles (vp/ml), the amount of polymer molecules needed was calculated for each specific molecules of polymer (pol):vp ratio. The volume of polymer stock was calculated taking into account the polymer stock concentration and the polymer molecular weight (MW). The calculated volume of polymer stock was diluted in PBS to a final volume of 100 µl (PS). Finally, the PS and VS solutions were mixed by adding PS to VS and pipetting up and down slowly at least 10 times. Samples were incubated 30 min at room temperature to enable the electrostatic interactions and used fresh. In order to prepare SAG101 samples, AdNuPARmE1A was coated at 4 × 10^6^ pol:vp ratio, and the polymer used is a 65:35 w/w mixture of C6CR3 and C6PEGCR3.

The average size, surface charge (Z-potential), and polydispersity index (PDI) of naked and coated virus preparations were determined using the Dynamic Light Scaterring (DLS) (ZetaSizer Nano SZ, Malvern Instruments Ltd, United Kingdom, 4-mW, 633nm laser) technique, at a scattering angle of 173º. Size distribution of different preparations were also measured using Nanoparticle Tracking Analysis (NTA) (Nanosight, Malvern Instruments Ltd, United Kingdom; 488 nm laser). The morphology and structure of coated virus preparations were studied by transmission electron microscopy (TEM) using the JEM 2011 and JEOL 1010 microscopes. Samples were negatively stained with 1% phosphotungstic acid solution (PTA) prior to imaging. TEM tomography was captured by rotating the sample from +60º to -60º. Images were collected every 2º of rotation. Three-dimensional representation of the resulting tomography constructed was performed using 3DMOD software.

### Gel-retardation assay

Twenty microliters containing 1 × 10^10^ VPs of naked Ad, C6CR3Ad, and CPEGAd were loaded onto a 1% (w/v) agarose gel containing ethidium bromide and electrophoresed at 80 V for 30 min in 1 × TAE buffer at pH 8.0 [10.0 mM Tris/HCl (pH 7.6), 1% (v/v) acetic acid, and 1.0 mM EDTA (pH 8.0)]. Gel images were acquired using a UV transilluminator.

### STORM imaging

Virus formulations were physically adsorbed onto flow chamber surfaces assembled from a glass slide and a coverslip (24mm x 24 mm, thickness 0.15 mm) separated by double-sided tape. Unbound particles were removed by washing excess sample with PBS. Finally, STORM buffer was fluxed into the chamber before imaging. Images were acquired using NIS-Elements software in Nikon Eclipse Ti microscope. Cy5-labelled polymer was imaged with a 647-nm laser (160 mW), taking 21,000 frames per image. DyLight 550 labelled viruses were imaged with a 561-nm laser (80mV) taking 20,000 frames per image.

Fluorescence images were collected as previously described 57. Briefly, images were collected using a Nikon 100x, 1.49 NA oil immersion objective and passed through a quad-band pass dichroic filter (97335 Nikon). Images were acquired onto a 256×256 pixel region (pixel size 0.16 µm) of a Hamamatsu 19 ORCA-Flash 4.0 camera at 10 ms integration time. Images were reconstructed using the STORM module of the NIS element Nikon software. Intensity threshold for both channels was settled at 250, and trace length values at minimum 1 and maximum 5.

### Infectivity studies

Infectivity studies were performed using the AdGFPLuc replication-defective virus. AdGFPLuc was tested naked or coated with different coating formulations. Viral samples were diluted with DMEM supplemented with 10% FBS in order to reach the desired experimental concentration. For nAbs pre-incubated conditions the culture media was also supplemented with a 10^4^ dilution of anti-Ad5 polyclonal antibody (ab6982; Abcam). Samples were incubated at RT during 30 min before infection. 15.000 PANC-1 cells/well in a 96 multi-well plate were infected at MOI 50. At four h post-infection, wells were washed, and fresh culture medium was added into each well. Forty-eight h after infection, cells were trypsinized and analyzed using Attune NxT Flow cytometer in order to determine the percentage of GFP-positive cells.

Infectivity studies in CAR+ (A549) and CAR- (MCF-7) were carried out infecting 5.000 cells/well with naked Ad and CPEGAd with increasing MOIs ranging from 0 to 4,500. At four h post-infection, wells were washed, and fresh culture medium was added into each well; at 48 h post infection, cells were analyzed by flow cytometry to determine the percentage of GFP-positive cells. The resulting transduction curves were used to extrapolate the MOI needed to produce 15% GFP+ cells.

### Stability test

Samples were coated and maintained in PBS at 37ºC. At each time-point, samples were diluted in DMEM 10% FBS and 15.000 PANC-1 cells/well were infected at MOI 50. After 48h, cells were analyzed by flow cytometry in order to determine the number of GFP+ cells in an Attune NxT Flow cytometer. Results are shown as %GFP + cells in comparison with cells infected with freshly prepared samples for each condition.

### Animal experiments

All animal procedures met the guidelines of European Community Directive 86/609/EEC and were approved by the Institutional Committee on Animal Use, Comité de Experimentación Animal (CEEA) of the UB (Universitat de Barcelona) protocol number DTS9244. Animals were housed in plastic cages, and under controlled environmental conditions of humidity (60%), temperature (22ºC ± 2ºC), and light, with food and water ad libitum.

### Blood clearance

Seven-week-old C57BL/6J male mice were injected intravenously with naked or CPEGAd with a total dose of 1 × 10^10^ vp/animal. At 2 min and 10 min post-administration, blood samples were collected from the saphenous vein using EDTA treated capillaries. Blood DNA Isolation Mini Kit (Product # 46300, 46380, Norgen Bioteck Corp.) was used to extract DNA from 50 μl whole blood samples following the manufacturer protocol. A qPCR of the samples was performed using hexon specific primers Hexo01= 5′-GCCGCAGTGGTCTTACATGCACATC-3′ and Hexo02= 5′- CAGCACGCCGCGGATGTCAAAG-3′.

### Biodistribution studies

C57Bl/6J were used for biodistribution studies and BALB/c Athymic Nu/Nu, to study the tumor-to-liver biodistribution. PANC-1 cells (1 × 10^6^) were injected subcutaneously in BALB/c Nu/Nu mice in each flank using a 29G needle. Cells were prepared in Matrigel Matrix Basement Membrane HC (Corning) by 1:1 mixing cells in DMEM without antibiotics and supplements and Matrigel to a final volume of 100 μl. Tumor progression was analyzed by measuring tumor's volume using a digital caliper. When tumors reached 100 mm^3^, 1 × 10^10^ vp/animal (n=5-6) of naked AdGFPLuc and CPEG-coated AdGFPLuc was intravenously injected. The same virus doses were injected in C57Bl/6J mice.

Five days after injection, luciferase activity was analyzed *in vivo* and *ex vivo*. For *in vivo* measurements, luciferase activity was visualized and quantified in living animals using an *in vivo* bioluminescent imaging system (Hamamatsu Photonics). Briefly, the substrate firefly D-luciferin (PerkinElmer, Inc) was I.P. administered (16 mg/kg), and after 10 min, animals were anesthetized with a mixture of isofluorane and oxygen preparation. Mice were introduced into the capturing cage coupled to an inhaled anesthesia system, and images were captured and analyzed using Wasabi software (Hamamatsu Photonics). For ex vivo measurements, luciferase assay of organs and tumors were carried out. Briefly, different organs and tumors were mechanically homogenized in a cold potter with liquid nitrogen to obtain a fine powder. Powder was mixed with lysis buffer (cell culture lysis reagent, Promega) and incubated 15 min at 25ºC. Samples were centrifuged for 10 min, 16000 g at 4ºC, and supernatants were collected. Luciferase activity was quantified using the Luciferase Assay System Kit (Promega), and photon emission was measured in a Synergy HT luminometer (BioTek). Light emission was normalized to total protein levels. Protein concentration was determined with BCA protein assay (Pierce Biotechnology).

### Cell viability assay

PANC-1, MIA PaCa-2, and A549 cells (10.000 cells/well) were incubated with naked AdNuPARmE1A and SAG101 serially diluted from 0 to 20.000 vp/cell. Three days later, cell viability was measured and quantified by a colorimetric assay system based on the tretrazolium salt 3-(4,5-dimethylthiazol-2-yl)-2,5-diphenyl tetrazolium bromide (MTT; Roche Molecular Biochemicals), in accordance with the manufacturer's instructions. Absorbance was measured at 590 nm with background correction at 630 nm using a microplate reader. Results were expressed as the percent absorbance determined in treated wells relative to that in untreated wells. IC_50_ values were estimated from dose-response curves by standard non-linear regression GraphPad.

### Assessment of the antitumor efficacy in passively immunized mice

PANC-1 or MIA PaCa-2 tumors were established by injecting 2 × 10^6^ cells into the flanks of 7-week-old male BALB/c Nu/Nu mice. Cells were prepared as described above. Once tumors reached 100 mm^3^, mice were randomized (n = 8 per group), and two groups were passively immunized by injecting intraperitoneally 200 µl of neutralizing serum. The following day, animals were treated with a single intravenous injection of vehicle (saline) or 4 × 10^10^ vp of AdNuPARmE1A or SAG101. Tumor progression was analyzed by measuring the tumor volume (volume= [length × width^2^ × π]/6) using a digital caliper three times per week.

Neutralizing serums for passive immunizations were produced as follows. C57BL/6J naïve mice were immunized by injecting two doses of 3 × 10^10^ vp/animal of naked AdGFPLuc intravenously at day 0 and day 14. At day 21 after the first injection, blood was collected by intracardiac puncture and sera were obtained, heat inactivated, and pooled.

### *In vivo* toxicity study in mice

Blank, (Saline), Vehicle (saline, 0.9% glycerol, 6.2% DMSO) or 5 × 10^10^ vp/animal (low dose) or 7.5 × 10^10^ vp/animal (high dose) of AdNuPARmE1A or SAG101 were injected intravenously into the tail vein in 7-week-old immunocompetent BALB/C male mice (n = 3-7). Animals were weighed and examined daily for clinical signs of toxicity. At the indicated time-points, blood aliquots were collected for platelet count, cytokine determination, transaminases activity, and hematologic studies.

### Serum AST and ALT analysis

Blood samples were left 30 min at room temperature to induce blood clotting and then centrifuged (3500 rpm, 15 min). The obtained sera were stored at -80ºC. AST and ALT analysis were conducted at the clinical biochemistry service of Hospital Clínic de Barcelona.

### Hematologic study

Blood samples for platelet cell count were collected using heparinized blood collection capillaries and platelets were manually counted using a hematocytometer diluting 25 µl of blood with lysis buffer (1% ammonium oxalate solution) to induce lysis of red blood cells (RBC). Hemograms were performed with whole blood collected by intracardiac puncture in EDTA tubes. Analyses were conducted at the Centre de Diagnostic Biomèdic (CDB) of Hospital Clínic de Barcelona.

### *In vitro* neutralization assay of serums produced *in vivo*

C57BL/6J naïve mice were immunized by injecting two 1 × 10^10^ VP/animal doses of naked Ad and CPEGAd formulations intravenously at day 0 and day 14. At day 21 after the first injection, blood was collected by intracardiac puncture and serums were obtained and heat inactivated. 5 × 10^5^ PFU/ml solution of AdGFPLuc was prepared and 50 μl of this solution was added to 96-well white plate wells. The serum sample was the diluted 10-fold with PBS, and 50 μl was added to each well. Samples were serially diluted by transferring 50 μl to the consecutive columns. After 30 min at RT, 1 × 10^5^ HEK293 cells were added to each well, and after 24 h, luciferase activity was quantified as described above. The ND50 values were calculated by determining the dilution which 50% neutralized the signal from the positive transduction control without serum.

### Evaluation of the Innate Immune Response

RAW264.7 macrophage cells were seeded in a 6-well-plate at 1 × 10^6^ cells/well in supplemented DMEM. After 24 h, culture medium was replaced with 1 ml of fresh serum-free medium containing 1000 vp/cell of AdNuPARmE1A, SAG101, the polymeric component without viral particles, or (as a vehicle control) the corresponding residual volume of DMSO used with SAG101. After 6 h, 500 µl of 10% FBS-containing medium was added to each well; after 48 h, culture media were collected from each well (avoiding cell collection). The concentration of IL6 was quantified using a microbead-based ELISA kit (Mouse Cytokine 10-Plex Panel; Invitrogen) on the Luminex™ 200™. IFN-γ, IL6 and TNF-α from mice sera were quantified with the same ELISA Kit.

### Statistical Analysis

Statistical analyses were carried out with Graph-Pad Prism (GraphPad Software). All error bars reported are SEM unless otherwise indicated. Pairwise comparisons were performed using one-way Student's *t*-tests and Tukey's multiple comparison test. Differences between groups were considered significant at *P* values below 0.05 (*P < 0.05, **P < 0.01, ***P < 0.001). Tumor progression data was compared between conditions using linear mixed effect in R v2.14.1 using the lme4 package.

## Supplementary Material

Supplementary figures.

## Abbreviations

Ad: adenovirus
ALT: alanine aminotransferase
AST: aspartate aminotransferase
CAR: Coxsackie adenovirus receptor
DLS: dynamic light scattering
dSTORM: direct stochastic optical reconstruction microscopy
MOI: multiplicity of infection
nAbs: neutralizing antibodies
NTA: nanoparticle tracking analysis
OM-pBAEs: oligopeptide-modified poly(β-amino ester)s
PDAC: pancreatic ductal adenocarcinoma
PEG: polyethylene glycol
PDI: polydispersity index
SAG101: AdNuPARmE1A coated with CPEG formulation
TEM: transmission electron microscopy
vp: virus particles
