# Supplementary material for: Oligopeptide-modified poly(beta-amino ester)s-coated AdNuPARmE1A: Boosting the efficacy of intravenously administered therapeutic adenoviruses
Source: Theranostics. 2020 Feb 3;10(6):2744–58. doi: 10.7150/thno.40902 (PMC7052890; doi:10.7150/thno.40902)
Supplement: Supplementary file 1 — Supplementary figures. [file thnov10p2744s1.pdf]

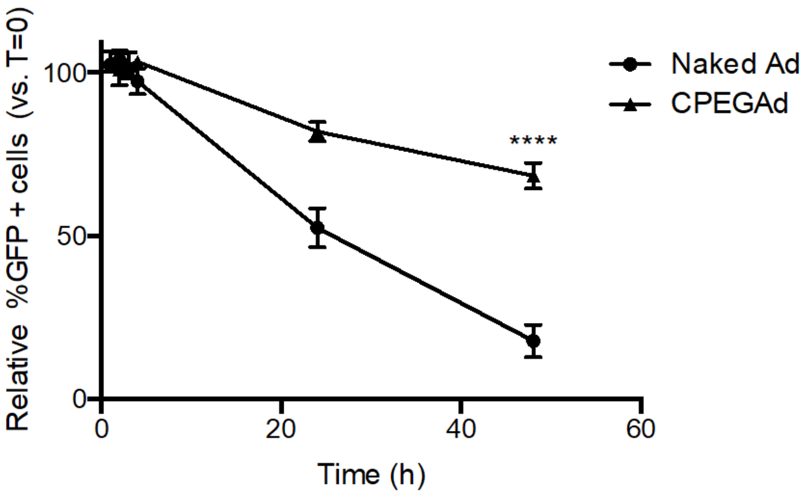

Figure S1. Stability of CPEGAd under physiological conditions. AdGFPLuc and CPEGAd were incubated at 37ºC in PBS for the indicated periods, after which they were tested in transduction assays in PANC-1 cells. Quantification of GFP-positive cells was performed at 48 hr post-transduction. Data show mean values ± SEM. **P* < 0.05, ***P* < 0.01, ****P* < 0.001, *****P* < 0.0001.

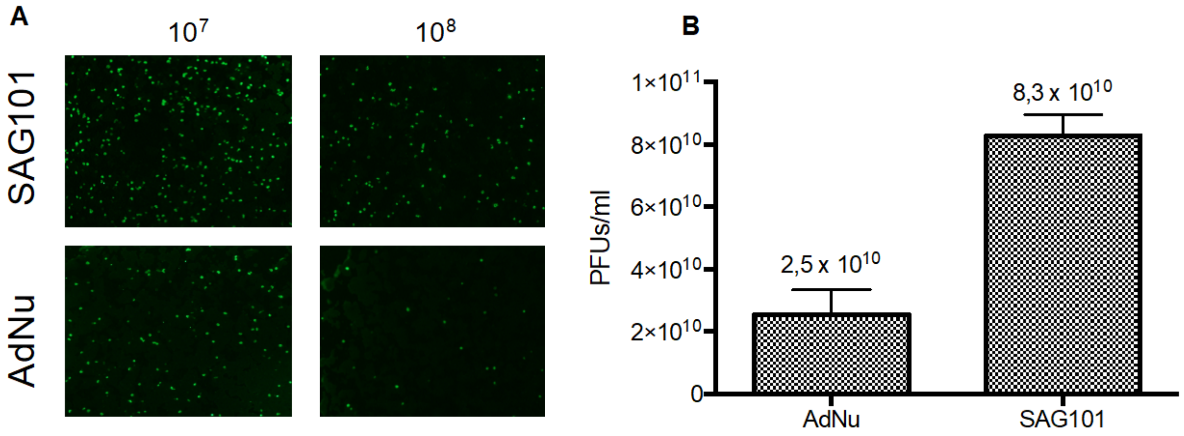

Figure S2. *In vitro* viral infectivity of SAG101. A Fluorescence microscopy images of HEXON immunostained PANC-1 cells infected at different SAG101 and AdNu virus sample dilutions. **B** Resulting virus titer obtained in PFUs/ml for each formulation.

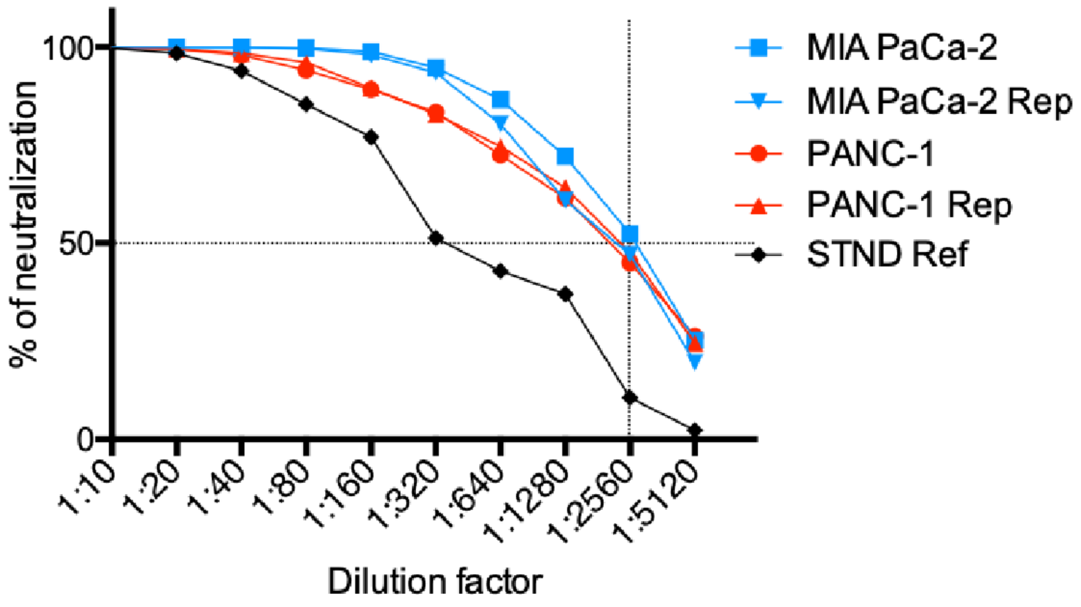

Figure S3. *In vitro* neutralization assay of Ad5 antiserums used for passive immunization *in vivo*. The neutralizing capacity of sera collected from naïve C57BL/6J mice after intravenous administration of two doses of naked Ad (1×10^{10} vp/animal) were administered at day 1 and day 14, and serum was collected at day 21 post-injection. The hatched lines indicate the ND50 of each sample. Each sample was analyzed twice (indicated by Rep) in triplicate.
